# The Involvement of Long Non-coding RNA and Messenger RNA Based Molecular Networks and Pathways in the Subacute Phase of Traumatic Brain Injury in Adult Mice

**DOI:** 10.3389/fninf.2022.794342

**Published:** 2022-03-04

**Authors:** Zhaoyu Yang, Xuexuan Li, Weikang Luo, Yao Wu, Tao Tang, Yang Wang

**Affiliations:** ^1^Institute of Integrative Medicine, Department of Integrated Traditional Chinese and Western Medicine, Xiangya Hospital, Central South University, Changsha, China; ^2^National Clinical Research Center for Geriatric Disorders, Xiangya Hospital, Central South University, Changsha, China

**Keywords:** traumatic brain injury, lncRNA and mRNA microarray, inflammation, immune response, subacute phase

## Abstract

Traumatic brain injury (TBI) is a complex injury with a multi-faceted recovery process. Long non-coding RNAs (lncRNAs) are demonstrated to be involved in central nervous system (CNS) disorders. However, the roles of lncRNAs in long-term neurological deficits post-TBI are poorly understood. The present study depicted the microarray’s lncRNA and messenger RNA (mRNA) profiles at 14 days in TBI mice hippocampi. LncRNA and mRNA microarray was used to identify differentially expressed genes. Quantitative real-time polymerase chain reaction (qRT-PCR) was employed to validate the microarray results. Bioinformatics analysis [including Gene Ontology (GO), the Kyoto Encyclopedia of Genes and Genomes (KEGG) pathway, lncRNA-mRNA co-expression network, and lncRNA-miRNA-mRNA network] were applied to explore the underlying mechanism. A total of 264 differentially expressed lncRNAs and 232 expressed mRNAs were identified (fold change > 1.5 and *P*-value < 0.05). Altered genes were enriched in inflammation, immune response, blood–brain barrier, glutamatergic neurological effects, and neuroactive ligand-receptor, which may be associated with TBI-induced pathophysiologic changes in the long-term neurological deficits. The lncRNAs-mRNAs co-expression network was generated for 74 lncRNA-mRNA pairs, most of which are positive correlations. The lncRNA-miRNA-mRNA interaction network included 12 lncRNAs, 59 miRNAs, and 25 mRNAs. Numerous significantly altered lncRNAs and mRNAs in mice hippocampi were enriched in inflammation and immune response. Furthermore, these dysregulated lncRNAs and mRNAs may be promising therapeutic targets to overcome obstacles in long-term recovery following TBI.

## Introduction

Traumatic brain injury (TBI) continues to be a prominent public health concern. In China, it is estimated that the mortality of TBI is approximately 13 cases per 100,000 people (Jiang et al., 2019). The pathological of TBI is complex, composed of primary and secondary injury. Primary injury is caused by direct mechanical insult. Secondary injury results from delayed metabolic, biochemical, and cellular changes initiated by the primary insult (Yang et al., 2019). Although many studies have been carried out in animal models and clinical patients with TBI, its prognosis is still poor (Xiong et al., 2013). Because the understanding, the underlying cellular and molecular mechanisms of TBI remain unclear.

Nowadays, high-throughput and bioinformatics technology have been broadly applied to identify the pathogenesis and progression of TBI (Banoei et al., 2018; Manek et al., 2018; O’Connell et al., 2020). It is clear that 90% of eukaryotic genomes are transcribed (Wilhelm et al., 2008), and only 1–2% of the genome encodes proteins (Birney et al., 2007), suggesting that the majority of transcripts represent non-coding RNA (ncRNA) (Zhong et al., 2016). Long non-coding RNA (lncRNA) are essential regulators of gene expression. They have been shown to mediate several cell cycle pathways, proliferation, apoptosis, and immune function (Hu et al., 2018; Zhao et al., 2018; Zhang P. et al., 2019; Zhuang et al., 2019). lncRNAs are also involved in various pathophysiological mechanisms of central nervous system (CNS) diseases, and targeting these lncRNAs can treat most CNS diseases (Ang et al., 2020; Kahaei et al., 2020; Tian et al., 2020), such as Alzheimer’s disease (Zhang et al., 2018), and ischemic stroke (Zhang et al., 2021). Furthermore, lncRNAs have specific expression profiles in brain tissue and can be used as potential independent prognostic molecular markers (Kadakkuzha et al., 2015; Ren et al., 2020). Several recent studies demonstrated the importance of lncRNAs in TBI. For instance, Patel et al. (2018) showed that lncRNA MALAT1 drives regenerative function and modulates inflammation network following TBI. Yang et al. (2019) observed expression characteristics of lncRNAs and mRNAs in human TBI tissues in the acute stage. Ren et al. (2020) investigated the expression profiles of lncRNAs and mRNAs in blood from TBI patients. Abundant evidence suggests that various lncRNAs are aberrantly expressed in mouse TBI tissues and can improve TBI by inhibiting the inflammatory response of microglia in the acute phase (Wang et al., 2017; Zhong et al., 2017; Patel et al., 2018). Although the transcriptomics analysis of acute TBI was well documented, the investigation of lncRNAs in subacute TBI remains necessary.

The TBI is a progressive disease with chronic consequences. In recent years, TBI has received widespread media attention as a risk factor for developing chronic traumatic encephalopathy (LoBue et al., 2019). Long-term disorder of consciousness in TBI has increased the burden on society and families. TBI can induce subtle alterations in molecular signaling, cellular structure, and function change in the subacute phase (Pearn et al., 2017). As a transition period, the subacute phase affects the long-term disorder of consciousness and determines the prognosis and development of TBI (Zheng et al., 2020). Unfortunately, studies of TBI in the subacute stage receive much less focus than those in the initial phase. The pathophysiologic processes in the subacute stage are still complicated, including inflammation, cell death, and cerebral atrophy (Kumar et al., 2015; Graham and Sharp, 2019; Witcher et al., 2021). The aberrant substance in the subacute stage might exit long; the disturbed substance could influence the prognosis and development of TBI (Zheng et al., 2020). Therefore, exploring the subacute process is vital to find optimal therapies and improve neurological outcomes after TBI.

In the present study, we focused on the expression signatures of lncRNAs and mRNAs in the subacute TBI. Differentially expressed (DE) lncRNAs and mRNAs in the hippocampus after subacute TBI in mice were screened by microarray. We applied a comprehensive analysis of both lncRNA and mRNAs expression in the hippocampus from controlled cortical impact (CCI) mice and compared it to sham group. Afterward, significantly altered mRNA-related biological functions and pathways were determined. Moreover, enrichment analyses with Gene Ontology (GO) and Kyoto Encyclopedia of Genes and Genomes (KEGG) were carried out to find DE genes via building a co-expression network. The relationship between lncRNAs and mRNAs was further analyzed with *cis*- and *trans*-regulation style and constructed the lncRNA-miRNA-mRNA networks.

## Materials and Methods

### Animals

Male adult C57BL/6 mice (25 ± 3 g, 8–10 weeks old) were individually housed and were allowed free access to water and food. Food was withheld overnight before surgery. The Experiment Committee of Central South University approved all animal procedures. Ethical guidelines by the ARRIVE (Animal Research: Reporting *In Vivo* Experiments) standards. All surgeries were performed under anesthesia, and all the efforts were made to minimize the suffering of mice.

### Controlled Cortical Impact Model

As previously reported, a controlled cortical impact model was conducted (Yang et al., 2010; Zhong et al., 2016). Briefly, the mice were anesthetized with 0.3% sodium pentobarbital (60 mg/kg) and positioned in a stereotaxic frame. A midline longitudinal incision was then performed, and the skin retracted and the skull exposed. A 4.0 mm-diameter craniotomy was made in the right parietal bone midway between bregma and lambda with the medial edge 1 mm lateral to the midline. Mice were impacted at 3.5 m/s with 80 ms dwell time and 1 mm depth using a TBI device (TBI-0310 Impactor, PSI, United States), which enables the application of standard contusion injury to the brain. The bone flap was discarded, and the scalp was sutured closed, surgical knots being used to secure the suture. Take care to keep the mice warm during the operation. The mice were then returned to their cages after recovering from anesthesia.

### The Modified Neurological Severity Score Test

The mNSS test was applied to evaluate post-traumatic neurological impairment, as previously described (Xing et al., 2016; Feng et al., 2017; Xiao et al., 2021). The eighteen-point mNSS consists of motor tests (six points), sensory tests (two points), beam balance tests (six points), reflexes absent, and abnormal movements (four points). The severity of the injury is directly graded on a scale of 0 (normal) to 18 (maximal deficit). In each group, five mice were examined to evaluate the neurological function scores on days 0 (post-injury), 1, 3, 7, and 14. The test was conducted by two investigators who were blind to the experimental groups. An average value was calculated for each mouse on each day of testing.

### Corner Turn Test

The corner turn test was conducted as previously described (Li et al., 2019, 2020; Xiao et al., 2021). Mice would proceed into a corner with an angle of 30° degrees. To exit the corner, individual mice could turn left or right, and the direction taken was then recorded. This was repeated ten times per animal, with at least 30 s between trials. The percentage of right turns was calculated. The right turn rate in normal mice was about 50%, while the injured ones would have a higher percentage.

### Experimental Groups and Array Data Production

The CCI group received all surgical procedures and impact injuries. The sham group was subjected to surgical procedures except for impact injury. Ten hippocampi (five from the CCI group and five from the sham group) were obtained to microarray.

Each mice’s ipsilateral hippocampus (right) was dissected for microarray analysis and lysed using TRIzol reagent (Invitrogen, Thermo Fisher Scientific Corporation, Carlsbad, CA, United States). The total RNA was extracted in line with the manufacturer’s protocol. NanoDrop ND-1000 measured RNA quantity and quality. Standard denaturing agarose gel electrophoresis was applied to assess RNA integrity. Arraystar Mouse LncRNA Microarray V4.0 is designed to systematically profile lncRNAs along with the entire set of protein-coding mRNAs. The sample labeling and array hybridization were performed based on the Agilent One-Color Microarray-Based Gene Expression Analysis protocol (Agilent Technology). Firstly, an mRNA isolation kit (mRNA-ONLY™ Eukaryotic mRNA Isolation Kit, Epicentre) removes rRNA and purifies mRNA from total RNA. Then, according to a random priming method (Arraystar Flash RNA Labeling Kit, Arraystar), each sample was amplified and translated into fluorescent cRNA along the entire length of the transcripts without 3′ bias. Using the RNeasy Mini kit (Qiagen), purify the labeled cRNAs. NanoDrop ND-1000 was used to observe the concentration and particular activity of the labeled cRNAs. Each labeled cRNA (1 μg) was fragmented via adding 10 × Blocking Agent (5 μl) and of 25 × Fragmentation Buffer (1 μl), and then the mixture was heated (at 60°C, 30 min). It was finally diluting the labeled cRNA by adding 25 μl 2 × GE Hybridization buffer. Hybridization solution (50 μl) was distributed into the gasket slide and fabricated to the lncRNA expression microarray slide. The slides were incubated for 17 h in an Agilent Hybridization Oven (65°C). Use the Agilent DNA Microarray Scanner to clean, fix, and scan the hybridized arrays.

### Differential Expression Analysis for Long Non-coding RNAs and Messenger RNAs

The array images were obtained and analyzed by Agilent Feature Extraction software (version 11.0.1.1). The GeneSpring GX v12.1 software package (Agilent Technologies) was applied to quantile normalize and subsequent data process. LncRNAs and mRNAs with flags in Present or Marginal (“All Targets Value”), following quantile normalization of the raw data, were selected for further data analysis. We identified differentially expressed lncRNAs and mRNAs with statistical significance between the two groups through fold change > 1.5 and *P*-value < 0.05.

### Quantitative Real-Time Polymerase Chain Reaction Validation

The lncRNAs and mRNAs expression levels from microarray data were validated by qRT-PCR. In brief, the hippocampi from two groups were lysed using TRIzol reagent (Invitrogen). Then the total RNA was extracted in line with the manufacturer’s protocol. Reverse transcription to cDNA used a SuperScript™ III Reverse Transcriptase kit (Invitrogen, 18080-044). qRT-PCR was performed using 2 × PCR master mix (Arraystar, AS-MR-006-5) in a Thermal Cycler Dice Real-Time System II. The primer sequences are displayed in Table 1. All experiments were carried out in triplicate, and the lncRNAs and mRNAs expression levels were normalized to their internal control. Using the ΔCt method calculated the *C*t value for each sample, and the results were shown as 2^–ΔΔ*C*t^.

**TABLE 1:** Reverse-transcription polymerase chain reaction primers.

Name	Primers	Length (bp)
GAPDH (mouse)	F:5′ CACTGAGCAAGAGAGGCCCTAT3′ R:5′ GCAGCGAACTTTATTGATGGTATT3′	144
Clec5a	F:5′ CTTCTCCTTCTCCGAATCACC 3′ R:5′ GGTAACATTGCCATTGAACAC3′	208
Hsh2d	F:5′ TCCTCGGCTGGACTGGTTTG3′ R:5′ CTGTGGCTGACTCGGATAAGAA3′	153
Hagh	F:5′ CCTGTCAACACCCTGCCATAC3′ R:5′ CTTCCCACAGCCAGCAACAAAC 3′	118
ENSMUST00 000231680	F:5′ AATTTCAAGCCAAGATCCCACTG 3′ R:5′ TGACAGGTTCTCTTTGGAGTGC 3′	136
ENSMUST000 00229478	F:5′ GGTGAAGAGGAAGGCAGATGATG 3′ R:5′ CGTCAGTCTTTCCAAGTCCGTGT 3′	106
ENSMUST000 00209771	F:5′ GGAGTTGGGCAGAAGTGAGTG 3′ R:5′ GGAGACCTTTCTGTATCGTGGA 3′	137

### Functional and Pathway Enrichment Analysis

All differentially expressed mRNAs were selected for GO and KEGG pathway analyses to investigate the possible role of mRNAs and lncRNAs co-expressed with these mRNAs. We were using Metascape software^1^ to obtain GO enrichment. The DE mRNAs and their enrichment in different pathways were mapped using the KOBAS 2.0 software^2^. Likewise, we performed GO and KEGG enrichment analyses of DE lncRNA co-expressing differential mRNAs using DAVID (v6.8) (Huang da et al., 2009a,b). A *P*-value cutoff <0.05 was used to determine the enriched biological processes and pathways.

#### Long Non-coding RNA-Messenger RNA Co-expression Network Construction

Pearson’s correlation test was conducted to calculate the correlation between the expression levels of each DE lncRNAs and DE mRNAs. The correlation coefficient >0.8 and *P*-values < 0.05 were defined as the DE lncRNA--DE mRNA pairs co-expressed. Cytoscape software (version v3.7.1^3^) was used to visualize the gene co-expression network.

#### *Cis*- and *Trans*-Regulatory Long Non-coding RNAs Establishment

Long non-coding RNAs functions by acting on protein-coding genes via *cis*-acting elements and *trans*-acting factors. The closest coding mRNAs to lncRNAs 100 kb upstream and downstream were screened, and their associations with lncRNA were analyzed using the FEELnc (Wucher et al., 2017) software.

### Long Non-coding RNA-miRNA-Messenger RNA (ceRNA) Regulatory Networks Acquirement

We establish lncRNA-miRNA interactions using DIANA-LncBase^4^ (Paraskevopoulou et al., 2016). The miRNA-mRNA interactions were expected by miRDB^5^ (Liu and Wang, 2019) with a target score of more than 80. Accordingly, a ceRNA network was established by Cytoscape software (version v3.7.1, see Text Footnote 3).

### Statistical Analysis

Statistical analysis was performed using the SPSS 22.0 software (IBM Corp., Armonk, NY, United States). The significant differences in expression levels between TBI and sham groups were tested using a two-tailed Student’s *t*-test. The GO terms and enrichment of pathway identifiers’ significance in the DE mRNAs were evaluated using Fisher’s exact test. *P*-value < 0.05 was considered statistically significant.

## Results

### Traumatic Brain Injury Induced Neurological Deficits

**FIGURE 1:**
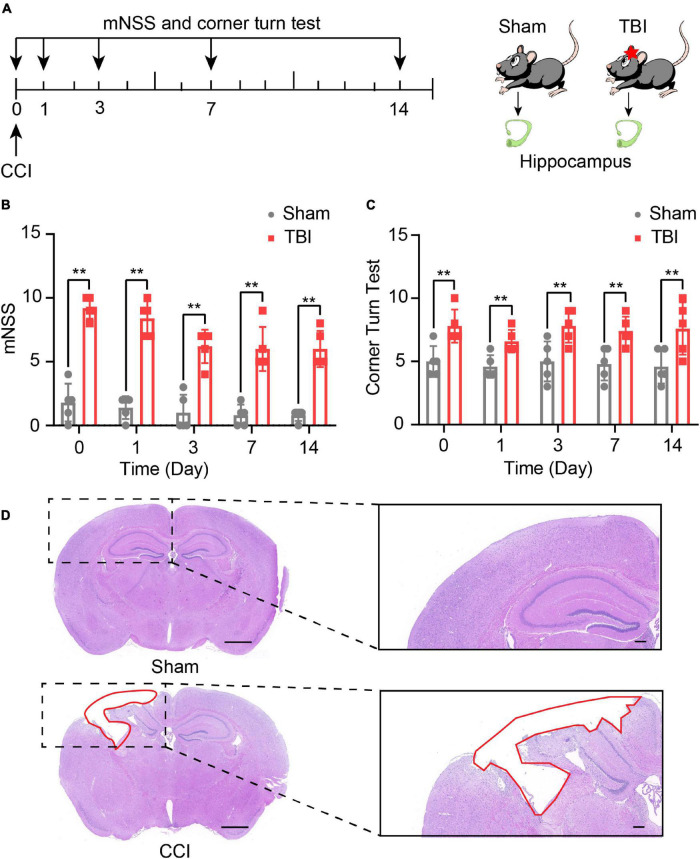
Traumatic brain injury (TBI)-induced neurological and brain tissue deficits. **(A)** Overview of experimental design and timeline for the experiment. Neurological deficits were evaluated with the modified neurological score (mNSS) and corner turn test from day 0 to day 14. The hippocampus was obtained on day 14. Using Modified neurological severity score (mNSS) test **(B)** and corner turn test **(C)** assess the neurological function of sham and TBI groups. The data are displayed as the mean ± SD, *n* = 5 per group. **P < 0.01, two-way ANOVA and two-tailed unpaired Student’s *t*-test. **(D)** The pathological analysis of sham and TBI groups (scar bar = 1,000 μm).

The experimental scheme, including the outcomes assessment and the experimental designs, is illustrated in Figure 1A. mNSS score was used to detect neurological deficits in motor, sensor, reflex, and equilibrium sense. The mNSS scores of the sham group and CCI group were summarized in Figure 1B. Compared with the sham group, the CCI group received higher scores at day 0 (*P* < 0.01), day 1 (*P* < 0.01), day 3 (*P* < 0.01), day 7 (*P* < 0.01), and day 14 (*P* < 0.01, Figure 1B). In the corn turn test, the results could distinguish the degree of motor deficits of mice in the CCI group and a sham group from day 0 to day 14 (*P* < 0.01, Figure 1C). These findings suggest that CCI promotes neural functional deficit. H&E staining was performed on brain tissues 14 days after CCI. Figure 1D shows that CCI caused brain lesions compared with the sham group. All results demonstrate the success of our model.

### Identification of Differentially Expressed Long Non-coding RNAs and Messenger RNAs

**FIGURE 2:**
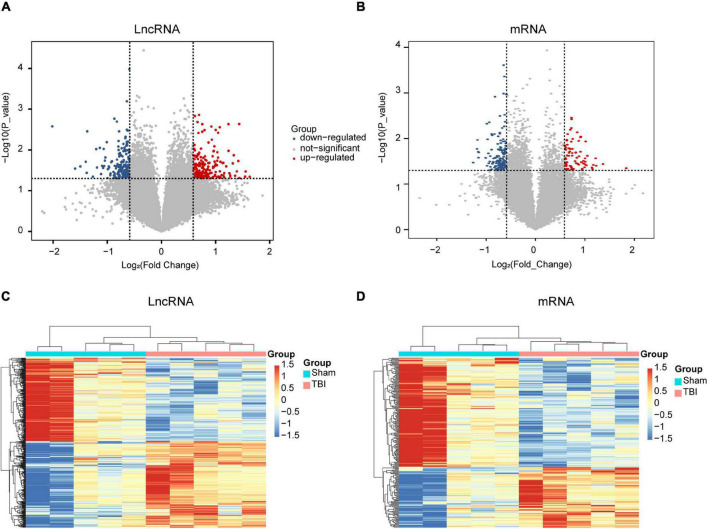
Expression profiles of DE lncRNAs and mRNAs in TBI and sham groups. Volcano maps of differentially expressed lncRNAs **(A)** and mRNAs **(B)**. The *x*- and *y*-axes have values of log_2_ (fold change) and -log_10_ (*P*-value). With a fold change > 1.5 and a *P*-value < 0.05, red/blue dots indicate statistically considerable differentially expressed lncRNAs and mRNAs (red depicts elevated expression while blue indicates decreased expression). No differentially expressed lncRNAs and mRNAs are indicated by gray dots. The hierarchical cluster analysis generated heat maps of DE lncRNAs **(C)** and mRNAs **(D)** (fold change > 1.5 and *P* < 0.05). DE, differentially expressed.

To understand the underlying mechanism of TBI, we performed lncRNAs and mRNAs microarray analysis. A total of 264 lncRNAs (115 upregulated and 149 downregulated) were differentially expressed (DE) in the CCI group compared with the sham group (Figure 2A). The top five lncRNAs with the most considerable fold changes are shown in Table 2. A total of 232 mRNAs were DE in the CCI group compared to the sham group. Eighty-three mRNAs were upregulated among these genes, and 149 mRNAs were downregulated (Figure 2B). Mitochondrial transcription termination factor 1 (Mterf1a), olfactory receptor 1241 (Olfr1241), Alanine-glyoxylate aminotransferase 2 (Agxt2), C-type lectin domain family five-member A (CLEC5A, also named as myeloid DAP12-associating lectin 1, MDL1), leukocyte immunoglobulin-like receptor member 4 (Lilr4b) are the top five upregulated mRNAs. Myosin light chain kinase 3 (Mylk3), Wnt family member 9b (Wnt9b), ankyrin repeat domain 29 (Ankrd29), hydroxyacylglutathione hydrolase (Hagh), collagen type X alpha 1 chain (Col10a1) are the top five downregulated mRNAs with the most extensive fold changes are shown in Table 3.

**TABLE 2:** Top five upregulated and downregulated lncRNAs in traumatic brain injury (TBI).

LncRNA	*P*-value	FC	Log2FC
**Top five upregulated lncRNAs**			
AK131807	0.0454477849023	3.0916903	1.628395809
ENSMUST00000209122	0.0338419474291	2.9383003	1.55498185
ENSMUST00000133329	0.0451465310322	2.9162158	1.544097483
ENSMUST00000211381	0.0488416334352	2.7105293	1.438574602
ENSMUST00000226858	0.0023163199043	2.705388	1.435835517
**Top five downregulated lncRNAs**			
AK082031	0.0026519572753	4.0457775	−2.01641698
ENSMUST00000138377	0.0286826317581	3.0186735	−1.593914723
ENSMUST00000161862	0.0254767731204	2.8211169	−1.496266449
ENSMUST00000231680	0.0195511489491	2.6351251	−1.397871454
TCONS_00016113	0.0366361773511	2.6011556	−1.379152703

**TABLE 3:** Top five upregulated and downregulated mRNAs in TBI.

mRNA	*P*-value	FC	Log2FC
**Top five upregulated mRNAs**			
Mterf1a	0.044884317	3.571796	1.836649685
Olfr1241	0.036293872	2.577447	1.365942762
Agxt2	0.039604876	2.3412416	1.227273818
Clec5a	0.027231169	2.324762	1.217083026
Lilr4b	0.0379632	2.2516481	1.170981373
**Top five downregulated mRNAs**			
Mylk3	0.033775	2.3989488	−1.262402367
Wnt9b	0.016916	2.2740471	−1.185262136
Ankrd29	0.010745	2.2602649	−1.176491864
Hagh	0.01949	2.2350134	−1.160283481
Col10a1	0.026288	2.2263319	−1.154668685

To further examine these differentially expressed genes, we constructed a hierarchical clustering map. The five TBI groups clustered together in one group were primarily distinct from the sham group. Overall, changes in the state from sham group to TBI group were also separated by differences in expression profiles of lncRNAs and mRNAs (Figures 2C,D). These results show that potential dynamic interactions between coding RNAs and lncRNAs may remold the whole transcriptomic landscape during the pathological process of TBI.

### Validation of Differentially Expressed Long Non-coding RNAs and Messenger RNAs Expression Levels

**FIGURE 3:**
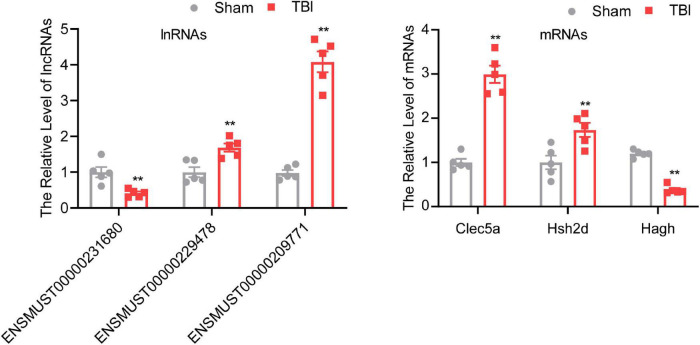
Validation of different expressed mRNAs and lncRNAs. The data are defined as the mean ± SD, *n* = 5 per group. **P < 0.01, One-way ANOVA, and two-tailed unpaired Student’s *t*-test.

To confirm the result of the microarray, several lncRNAs and mRNAs were detected by qRT-PCR. One downregulated (ENSMUST00000231680) lncRNA, two upregulated (ENSMUST00000229478 and ENSMUST00000209771) lncRNAs, one downregulated (Hagh) mRNA, and two upregulated (Clec5a and Hsh2d) mRNAs were chosen for qRT-PCR (Figure 3). The results of qRT-PCR were consistent with the microarray results. Three lncRNAs and three mRNAs were differentially expressed in the TBI group compared with the sham group (*P* < 0.01), indicating the reliability of the microarray data.

### Functions Examination of Differentially Expressed Messenger RNAs

**FIGURE 4:**
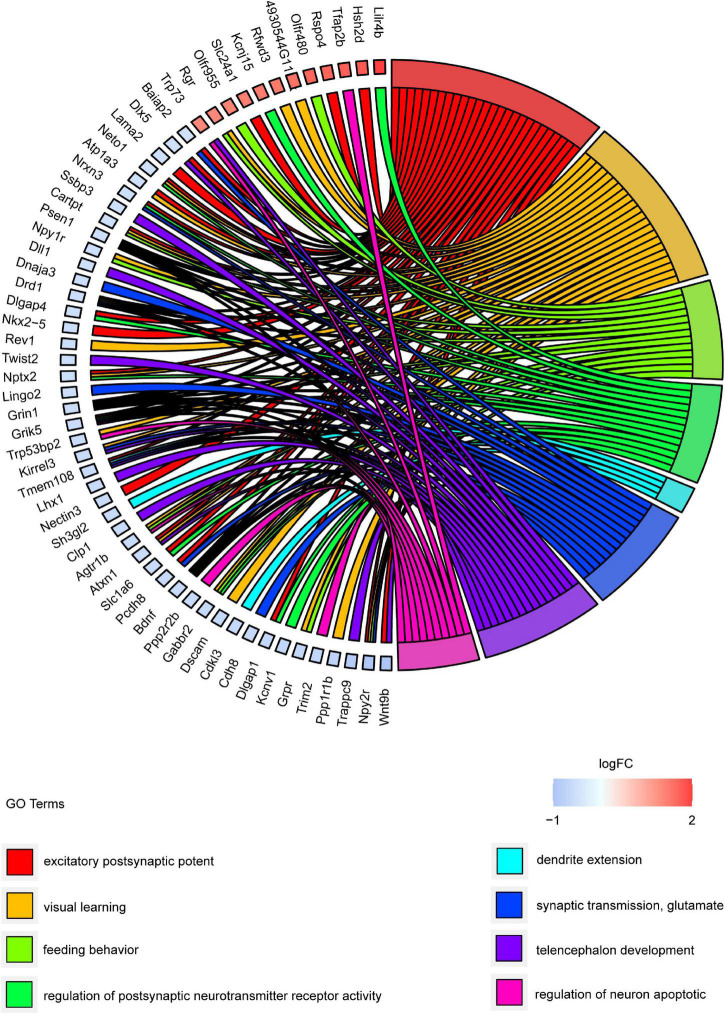
GO cluster plot showing a chord dendrogram of clustering of the expression spectrum of significantly DE genes. DE, differentially expressed; GO, Gene Ontology.

**FIGURE 5:**
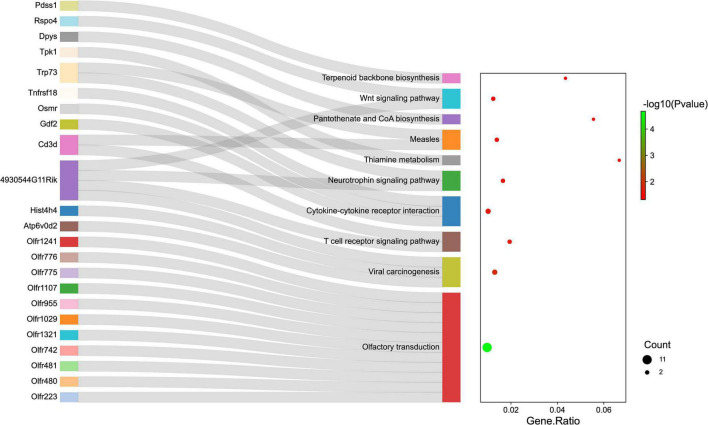
The KEGG enrichment analysis of up-regulated genes. The *y*-axis represents KEGG-enriched terms. The *x*-axis represents the Gene Ratio. The dot size stands for the number of genes under a specific time. The color of the dots represents the *P*-value. The left cluster plot shows a chord dendrogram of clustering the expression spectrum of significantly DE genes. DE, differentially expressed; KEGG, Kyoto Encyclopedia of Genes and Genomes.

**FIGURE 6:**
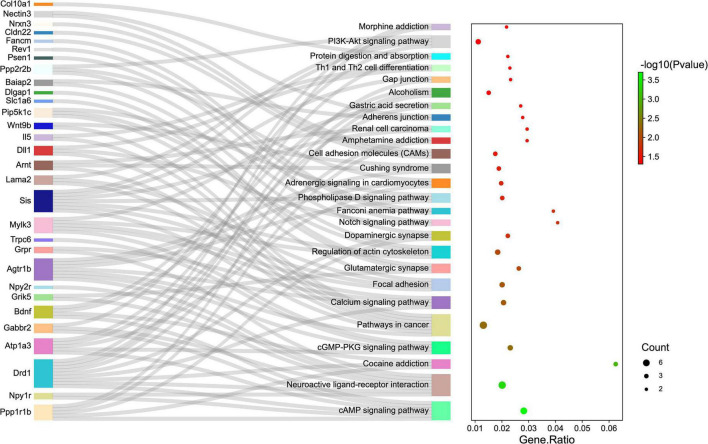
The KEGG enrichment analysis of down-regulated genes. The *y*-axis represents KEGG-enriched terms. The *x*-axis represents the Gene Ratio. The size of the dot demonstrates the number of genes under a specific term. The color of the dots represents the *P*-value. The left cluster plot shows a chord dendrogram of clustering the expression spectrum of significantly DE genes. DE, differentially expressed; KEGG, Kyoto Encyclopedia of Genes and Genomes.

Gene ontology and KEGG pathway analyses were conducted to understand the functions of the 232 DE mRNAs. Through the GO analysis of DE mRNAs, they were found to be mainly enriched as follows: excitatory postsynaptic potent (GO:0060079), visual learning (GO:0008542), feeding behavior (GO:0007631), regulation of postsynaptic neurotransmitter receptor activity (GO:0098962), dendrite extension (GO:0097484), synaptic transmission, glutamate (GO:0035249), telencephalon development (GO:0021537), and regulation of neuron apoptotic (GO:0043523) (Figure 4 and Table 4). KEGG pathway was applied with the downregulated and upregulated DE mRNAs separately. KEGG pathway analysis revealed that the upregulated DE mRNAs were mainly associated with inflammatory and immune responses, according to KEGG pathway analysis (Figure 5 and Table 5). In addition, the downregulated DE mRNAs exhibited a strong association with the blood–brain barrier, glutamatergic neurological effects, neuroactive ligand-receptor, inflammation, and immune cells (Figure 6 and Table 6).

**TABLE 4:** GO enrichment analysis of DE mRNAs.

ID	Term	Counts	*P*-value
GO:0060079	Excitatory postsynaptic potent	26	4.62915E-08
GO:0008542	Visual learning	22	8.2669E-07
GO:0007631	Feeding behavior	13	4.17736E-06
GO:0098962	Regulation of postsynaptic neurotransmitter receptor activity	13	1.38247E-05
GO:0097484	Dendrite extension	4	1.47726E-05
GO:0035249	Synaptic transmission, glutamate	6	1.49632E-05
GO:0021537	Telencephalon development	9	2.12315E-05
GO:0043523	Regulation of neuron apoptotic	9	4.71992E-05

*GO, Gene Ontology; DE, differentially expressed.*

**TABLE 5:** KEGG enrichment analysis of DE mRNAs (up-regulated).

Term	Counts	P-value	Genes
Olfactory transduction	11	2.11E-05	Olfr223/Olfr480/Olfr481/Olfr742/Olfr1321/Olfr1029/Olfr955/Olfr1107/Olfr775/Olfr776/Olfr1241
Viral carcinogenesis	3	0.01238	Atp6v0d2/Hist4h4/4930544G11Rik
T cell receptor signaling pathway	2	0.019686	Cd3d/4930544G11Rik
Cytokine-cytokine receptor interaction	3	0.023579	Gdf2/Osmr/Tnfrsf18
Neurotrophin signaling pathway	2	0.026423	4930544G11Rik/Trp73
Thiamine metabolism	1	0.032236	Tpk1
Measles	2	0.036176	Cd3d/Trp73
Pantothenate and CoA biosynthesis	1	0.038166	Dpys
Wnt signaling pathway	2	0.044625	4930544G11Rik/Rspo4
Terpenoid backbone biosynthesis	1	0.047968	Pdss1

*KEGG, Kyoto Encyclopedia of Genes and Genomes; DE, differentially expressed.*

**TABLE 6:** KEGG enrichment analysis of DE mRNAs (down-regulated).

Term	Counts	*P*-value	Genes
cAMP signaling pathway	6	0.000199	Ppp1r1b/Npy1r/Drd1/Atp1a3/Gabbr2/Bdnf
Neuroactive ligand-receptor interaction	7	0.000435	Npy1r/Grik5/Drd1/Npy2r/Agtr1b/Grpr/Gabbr2
Cocaine addiction	3	0.000976	Bdnf/Ppp1r1b/Drd1
cGMP-PKG signaling pathway	4	0.004741	Atp1a3/Trpc6/Mylk3/Agtr1b
Pathways in cancer	7	0.004797	Sis/Lama2/Arnt/Dll1/Il5/Wnt9b/Agtr1b
Calcium signaling pathway	4	0.007011	Grpr/Agtr1b/Mylk3/Drd1
Focal adhesion	4	0.007641	Pip5k1c/Sis/Lama2/Mylk3
Glutamatergic synapse	3	0.010167	Slc1a6/Dlgap1/Grik5
Regulation of actin cytoskeleton	4	0.010214	Pip5k1c/Baiap2/Sis/Mylk3
Dopaminergic synapse	3	0.015815	Ppp2r2b/Ppp1r1b/Drd1
Notch signaling pathway	2	0.016156	Dll1/Psen1
Fanconi anemia pathway	2	0.017375	Rev1/Fancm
Phospholipase D signaling pathway	3	0.020385	Pip5k1c/Sis/Agtr1b
Adrenergic signaling in cardiomyocytes	3	0.021448	Agtr1b/Atp1a3/Ppp2r2b
Cushing syndrome	3	0.024046	Agtr1b/Wnt9b/Arnt
Cell adhesion molecules (CAMs)	3	0.028879	Cldn22/Nrxn3/Nectin3
Amphetamine addiction	2	0.029206	Ppp1r1b/Drd1
Renal cell carcinoma	2	0.029206	Sis/Arnt
Adherens junction	2	0.032347	Baiap2/Nectin3
Gastric acid secretion	2	0.033964	Atp1a3/Mylk3
Alcoholism	3	0.041996	Bdnf/Ppp1r1b/Drd1
Gap junction	2	0.044304	Sis/Drd1
Th1 and Th2 cell differentiation	2	0.045212	Dll1/Il5
Protein digestion and absorption	2	0.047978	Col10a1/Atp1a3
PI3K-Akt signaling pathway	4	0.049661	Sis/Lama2/Bdnf/Ppp2r2b
Morphine addiction	2	0.049855	Gabbr2/Drd1

*KEGG, Kyoto Encyclopedia of Genes and Genomes; DE, differentially expressed.*

### Establishment of a Long Non-coding RNA-Messenger RNA Co-expression Network

**FIGURE 7:**
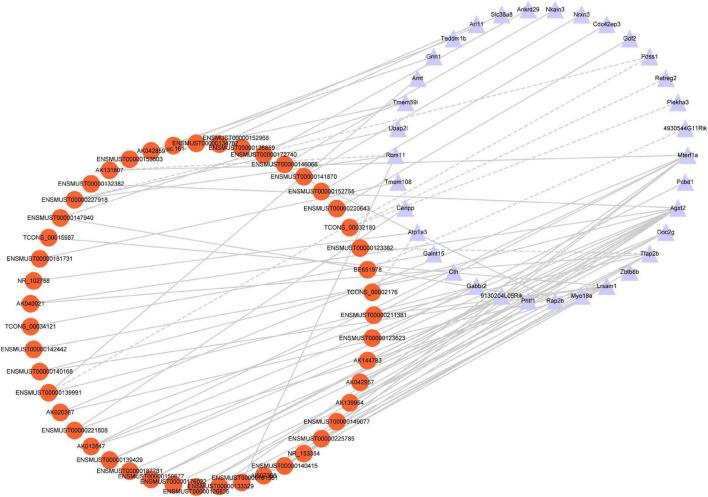
Co-expression network analysis. Red nodes represent lncRNAs; purple nodes represent mRNAs. A positive correlation is solid lines; a negative correlation is dashed lines.

To identify the correlation between DE lncRNAs and DE mRNAs, we performed the Pearson’s correlation (PC) test to calculate the correlation coefficients between lncRNAs and mRNAs. The correlation coefficient was >0.8, and *P*-value less than 0.05 was defined as the DE lncRNA-DE mRNA pairs co-expressed. We found 66 positive correlations DE lncRNA-DE mRNA pairs and eight negative correlations DE lncRNA-DE mRNA pairs (PC > 0.99 and *P* < 0.05) (Figure 7). The top five positive and negative correlations DE lncRNAs-DE mRNAs pairs are shown in Table 7. These results demonstrated that there is a close relationship between lncRNAs and mRNAs.

**TABLE 7:** Ten co-expressed lncRNAs and mRNAs.

lncRNAs	mRNAs	PC	*p*-value
**Top five positive correlation lncRNA-mRNA**			
ENSMUST00000211381	Mterf1a	0.9979	8.2910038617444E-11
ENSMUST00000123623	Pcbd1	0.9975	1.80313740532234E-10
AK144783	Agxt2	0.9974	2.05234532010625E-10
AK042957	Doc2g	0.9971	3.23390076397427E-10
AK139954	Tfap2b	0.9965	6.74601049681328E-10
**Top five negative correlation lncRNA-mRNA**			
TCONS_00002176	Atp1a3	−0.9954	1.92006614854314E-09
BE651978	4930544G11Rik	−0.9952	2.38482026028858E-09
ENSMUST00000227918	Pdss1	−0.9934	8.23914000478433E-09
AK131807	Rbm11	−0.9931	9.94895076628448E-09
ENSMUST00000123382	Tfap2b	−0.9928	1.13508663660169E-08

*PC, Pearson’s correlation coefficient.*

**FIGURE 8:**
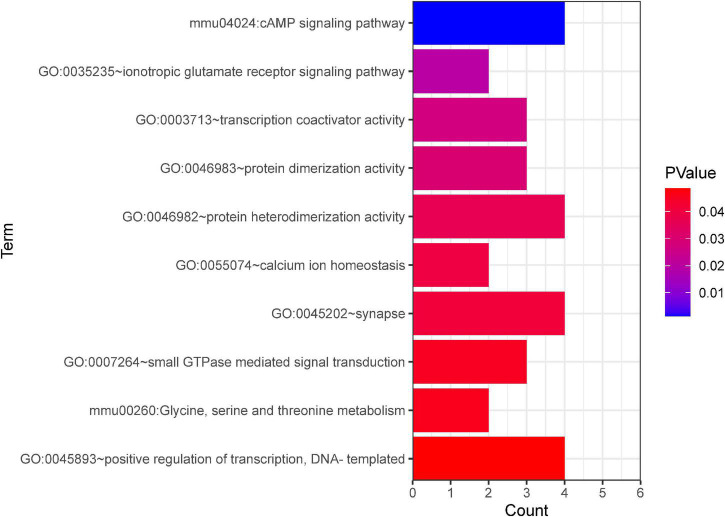
GO and KEGG pathway analyses based on the CNC network. The *x*-axis represents the number of genes. The color of the bars depicts the *P*-value. GO, Gene Ontology. KEGG, Kyoto Encyclopedia of Genes and Genomes.

Further, GO and KEGG enrichment analysis was applied using the co-expressed DE mRNAs. Several significant terms mainly were connected with neuron function, including cAMP signaling pathway (mmu04024), transcription coactivator activity (GO:0003713), calcium ion homeostasis (GO:0055074), synapse (GO:0045202), and glycine, serine, and threonine metabolism (mmu00260) (Figure 8).

### *Cis*- and *Trans*-Regulation of Long Non-coding RNAs in Traumatic Brain Injury

The function of lncRNAs on protein-coding genes was through *cis*-acting elements, and *trans*-acting factors-this trial explores how the DE lncRNAs may affect TBI. We predict the *cis*- and *trans*-regulated target genes of DE lncRNAs between TBI and sham groups combined with lncRNAs-mRNAs co-expression network. The closest coding mRNAs to lncRNAs 100 kb upstream and downstream were screened, and their associations with lncRNA were analyzed using the FEELnc (Wucher et al., 2017) software. Six *cis*-regulated correlation lncRNA-mRNA pairs (ENSMUST00000123623/Mpped2, ENSMUST00000 135180/Mpped2, ENSMUST00000140082/Ccdc187, ENSMUST 00000206530/Grik5, ENSMUST00000206530/Atpla3, and NR_045535/Lrsam1) and three *trans*-regulated correlation pairs (ENSMUST00000146256/Fdxr, TCONS_00032180/Shcbp1, and uc.233/Abcf2) exhibited in Table 8.

**TABLE 8:** *Cis*- and *trans*-regulated lncRNA and mRNA in TBI.

lncRNAs	Genome relationship	mRNAs	*P*-value	PC
ENSMUST00000123623	Upstream	Mpped2	2.37E-06	0.97266
ENSMUST00000135180	Upstream	Mpped2	7.02E-05	0.935458
ENSMUST00000140082	Downstream	Ccdc187	0.00341	0.823515
ENSMUST00000146256	Downstream	Fdxr	0.000395	−0.8995
ENSMUST00000206530	Upstream	Grik5	0.000482	0.894201
ENSMUST00000206530	Upstream	Atp1a3	0.000428	0.897388
NR_045535	Downstream	Lrsam1	2.07E-05	0.952698
TCONS_00032180	Downstream	Shcbp1	0.000254	−0.91033
uc.233+	Downstream	Abcf2	0.002837	−0.83189

*PC, Pearson’s correlation coefficient.*

### Competitive Endogenous RNA Network in Traumatic Brain Injury

**FIGURE 9:**
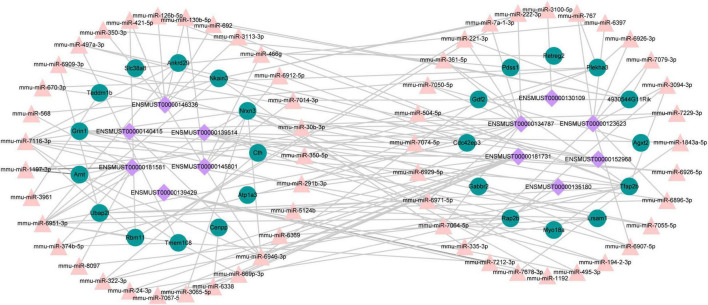
The ceRNA network between lncRNAs, mRNAs, and predicted miRNAs. Purple nodes, lncRNAs; green nodes, mRNAs; Pink nodes, miRNA.

A growing number of evidence supports that lncRNAs act as competitive endogenous RNAs for miRNAs and play a vital part in physiological and pathological processes (Salmena et al., 2011). We used DE lncRNA-DE mRNA co-expression data combined with predicted miRNA to construct the lncRNA-miRNA-mRNA regulatory network (Figure 9). To set up lncRNA-miRNA interactions, the likely MREs (miRNA Response Elements) in lncRNAs were predicted by lncBase. miRDB predicted the miRNA-mRNA interactions with a target score of more than 80. The interaction network included 12 lncRNAs, 59 miRNAs, and 25 mRNAs (Figure 9). Intriguingly, Nrnx3 was contained in the ceRNA networks. Nrnx3 exerts a vital function in synaptic formation and function (Feng et al., 2010). lncRNA ENSMUST00000181581 is connected to miRNAs (miR-6951-3p and miR-7116-3p) and Nrxn3. Therefore, ENSMUST00000181581 would compete with miR-6951-3p or miR-7116-3p to affect synaptic appearance and function during TBI.

## Discussion

Traumatic brain injury is a global health challenge due to its higher morbidity and mortality rates (Khellaf et al., 2019). Unluckily, there is still no valuable treatment for this devastating disorder. A better understanding of the precise molecular mechanisms after TBI will be critical for exploring potential new strategies for diagnosis and therapy. This study systematically analyzed the lncRNA-involved regulatory networks in mice brains after TBI based on microarray data of TBI and sham groups. To validate the reliability of the microarray results, three of the DE lncRNAs and DE mRNAs were verified with qRT-PCR. The GO and KEGG pathway enrichment analyses were used further to understand the potential biological functions of these DE mRNAs. We identified six *cis*- and three *trans*-regulated correlation pairs of lncRNA–mRNA from the co-expression analyses. Moreover, the lncRNA–miRNA–mRNA co-expression network was constructed. Our work provides a comprehensive, temporal explanation of molecular events attributed to the pathogenesis of TBI and uncovered functional lncRNAs regulatory networks in TBI.

We established a CCI mouse model as previously reported (Yang et al., 2010). mNSS and corner turn tests indicated that TBI induces neurological deficits in mice (Figures 1B,C). HE staining revealed that TBI leads to brain lesions (Figure 1D), which was quantitatively in good agreement with earlier reports (Xie et al., 2019). It was indicated that the animal model of CCI in our study was reliable. Then, we applied high-throughput microarray analyses to identify the expression profile of lncRNAs and mRNAs from the CCI and sham mice in the hippocampal tissue. Previously, Zhong et al. (2016) have investigated lncRNAs and mRNAs expression profiles in the cerebral cortex from a CCI mice model using the microarray. However, the hippocampus is widely recognized as a brain region vital for cognitive functions, such as spatial and episodic memory and learning (Castellano et al., 2017). Besides, the hippocampus is also considered one of the most sensitive areas of neuronal damage after TBI (Böttiger et al., 1998). Wang et al. (2017) have identified lncRNA and mRNA expression profiles in the hippocampus of fluid percussion injury rat model at 3 h post-injury. Day 14 is selected as an appropriate and reliable time point to represent a cognitive deficit in this study. Among the various hippocampal subfields, the dentate gyrus (DG), a hippocampal subfield, plays a vital role in learning and memory and is particularly susceptible to TBI due to its anatomical location (Hicks et al., 1993; Barha et al., 2011). TBI leads to neuronal loss in the hippocampal DG and induces cognitive deficits (Hicks et al., 1993). It has also been demonstrated that the level of Doublecortin (DCX, the marker of new neurons) in DG decreases in TBI animals over time (Herrick et al., 2006). Previously experiment found temporal changes in the immunity after TBI that diminished interleukin 12 expressions from day 14 after injury (Schwulst et al., 2013). Therefore, we focused on the hippocampus of mice to identify key molecules and related lncRNAs involved in cognitive deficits after 14 days following TBI.

The present research identified significantly deregulated lncRNAs and mRNAs (fold change > 1.5 and *P*-value < 0.05) in the hippocampal samples. Our results showed that 264 lncRNAs (Figures 2A,C) and 232 mRNAs (Figures 2B,D) were significantly differentially expressed between the two groups. In the present study, Mterf1a, Olfr1241, Agxt2, Clec5a, and Lilr4b were the five most significant up-regulated mRNAs, while Mylk3, Wnt9b, Ankrd29, Hagh, and Col10a1 were the five most significant down-regulated mRNAs in the CCI group. Olfactory receptors biomarkers are ectopically expressed in multiple brain regions, including the entorhinal-hippocampus system, which plays a vital role in memory formation and consolidation (Zhao et al., 2013). Olfr1241 is one of the olfactory receptors, downregulated in the olfactory bulb of the spinal cord injury (SCI) group mice at 8 h post-SCI (Lin et al., 2021). However, the relationship between Olfr1241 and TBI has not been documented. We have not found any previous research investigating the five up-regulated and down-regulated genes in TBI models of animals or humans. Their specific biological functions in TBI deserve to be further investigated. Further studies exploring the relevance of the above genes in the normal brain and the brain following TBI will better understand TBI’s biological mechanisms and insights into novel therapeutic targets for TBI. In this experiment, we also discovered the five most significantly up-regulated lncRNAs (AK131807, ENSMUST00000209122, ENSMUST00000133329, ENSMUST00000211381, and ENSMUST00000226858) and five most significantly down-regulated lncRNAs (AK082031, ENSMUST00000138377, ENSMUST00000161862, ENSMUST00000231680, and TCONS_00016113). LncRNAs are recognized as transcriptional regulators (Yoon et al., 2013; Long et al., 2017) that regulate protein-coding gene expression by *trans*- and *cis*-action mechanisms. However, no works have so far declared the association of these differentially expressed lncRNAs with TBI. Nonetheless, regarding the expression of mRNAs, we believe that these aberrantly expressed lncRNAs are also involved in the pathological process of TBI. Therefore, further surveys will be carried out to examine the function of these distinctively expressed lncRNAs in TBI.

Pathway enrichment analysis of the genes with differential expression suggested that inflammation and immune response (Figures 4, 5) may correlate closely with cognitive impairment following TBI. Recently, considerable evidence has shown that lncRNAs were related to cognitive function change (Faghihi et al., 2008; Shang et al., 2018), but the possible underlying mechanisms remain to be elucidated. As a regulatory factor, lncRNA was intended to be closely associated with inflammation, reactive oxygen species (ROS), and immune response (Chen et al., 2018; Xue et al., 2019; Wu et al., 2020). More importantly, many studies suggested that reducing neuronal inflammation or immune response could improve cognitive function after TBI (Denver et al., 2018; Tan and Norhaizan, 2019). In the context of cognitive deficits after TBI, we found that several inflammations and immune-related genes (Mterf1a, Trp73, Cd3d, Dll1, and Il5) had abnormal expression levels lncRNAs may potentially target. Thus, it implies that lncRNAs dysregulation may contribute to cognitive dysfunction following TBI insult by regulating neuronal inflammation or immune response.

Although the expression profile of lncRNAs has been shown to change extensively after TBI, the role of lncRNAs in TBI is still only partly known. In the present study, the correlations between specific lncRNAs and biological processes of TBI were connected by the CNC co-expression network. Gabbr2, Atp1a3, 4930544G11Rik, and Grin1 genes respond to TBI via the cAMP signaling pathway (Figure 7). A previous study demonstrated that the cAMP signaling pathway is downregulated after TBI (Atkins et al., 2007). In line with the prior research, our study identified the cAMP signaling pathway-related genes are down-regulated in TBI. The oxidative response is one of the significant hazards to TBI. Grin1 [*N*-methyl-D-aspartate receptor (NMDAR)] activation in human cerebral endothelial cells promoted intracellular oxidative stress (Sharp et al., 2005). The high co-expression level between AK042859 and Grin1 indicated that AK042859 might function as an oxidative response lncRNA in TBI. We also observed the co-expression between lncRNAs and other modules of genes essential for the pathophysiology of TBI, such as calcium ion homeostasis, regulation of transcript, synapse, and amino acid metabolism in TBI. These results may act as a framework for understanding the role of lncRNAs in TBI.

Currently, the potential functions of lncRNAs are investigated through their target genes by using *trans*- and *cis*-regulatory methods as previously described (Gao et al., 2017; Zhang F. L. et al., 2019). In the present study, we found six *cis*-regulated correlation pairs lncRNA-mRNA (ENSMUST00000 123623/Mpped2, ENSMUST00000135180/Mpped2, ENSMUST 00000140082/Ccdc187, ENSMUST00000206530/Grik5, ENS MUST00000206530/Atpla3, and NR_045535/Lrsam1) and three *trans*-regulated correlation pairs (ENSMUST00000146256/Fdxr, TCONS_00032180/Shcbp1, and uc.233/Abcf2). Previous studies demonstrated that metallophosphoesterase domain-containing 2 (Mpped2) is associated with inflammation (Schimunek et al., 2019), and overexpression of Mpped 2 will induce neuronal differentiation (Liguori et al., 2012). Ferredoxin reductase gene (Fdxr) has been known to be required for cell viability (Hwang et al., 2001), and loss of Fdxr function leads to a significant increase in ROS in neurodegenerative mitochondriopathy patients (Peng et al., 2017). Our findings indicated that these lncRNAs-target molecules might play an essential role in the cognitive impairment of mice after TBI.

Recently, competitive endogenous RNA (ceRNA) represents a novel layer of gene regulation that plays essential roles in the physiology and development of diseases (Salmena et al., 2011; Xu et al., 2015). lncRNAs affect the regulation of mRNA translation and stability primarily based on the ceRNA regulation mechanism of binding to miRNA. Increasing evidence demonstrated that the ceRNA regulatory network of lncRNA–miRNA–mRNA was important in TBI (Guo et al., 2017; Huang, 2018). Tmem108 (Transmembrane protein 108) is highly enriched in DG granule neurons, and its expression increased at the postnatal period critical for DG development (Jiao et al., 2017). Tmem108 expression is found as early as E8.5 in the central nervous system (Miller et al., 2008; Tang et al., 2010), and it plays a vital role in the central nervous system (Liu et al., 2007; Xu et al., 2016; Jiao et al., 2017). A Genome-wide association study (GWAS) found that TMEM108 is a susceptibility gene of psychiatric disorders, including schizophrenia, bipolar disorder, and major depression disorder (Neale et al., 2010; Consortium, 2011; Consortium et al., 2013; Ripke et al., 2013). In our study, Tmem108 was down-regulated in the CCI group. In the ceRNA network, lncRNA ENSMUST00000181731 connected with miR-669p-3p and target gene Tmem108. However, we have not found any previous study examining the relationship of ENSMUST00000181731, miR-669p-3p, and Tmem108 in TBI models of animals or humans. Hence, their specific biological functions in TBI deserve to be further investigated.

The results of our study are somewhat different from a previous study (Zhong et al., 2016; Wang et al., 2017). First, rats and mice are different species, with natural differences between them. Second, in the study of Wang et al. (2017), experiments were performed 3 h post-injury in rats. In contrast, the experiments in our study were performed 14 days post-injury in mice. We are concentrated on the alteration of molecular mechanisms in the subacute stage of TBI. TBI can induce subtle alterations in molecular signaling, cellular structure, and function change in the subacute phase (Pearn et al., 2017). As a transition period, the subacute phase affects the long-term disorder of consciousness and determines the prognosis and development of TBI (Zheng et al., 2020). Third, in this study, the hippocampus was applied to investigate the underlying mechanism. Therefore, although our results provide a clue for further research of potential mechanisms of gene regulation in TBI, we must verify and investigate the related genes.

Our data provide a bioinformatics analysis of genes and pathways that may be involved in the pathological mechanisms of TBI. Nevertheless, further studies are still needed to investigate their mechanism in the occurrence and development of TBI. Several lncRNAs are regarded as transcriptional regulators that regulate coding gene expression by *cis*- or *trans*-mechanisms. Unfortunately, minimal studies have discussed the association of these distinctively expressed lncRNAs with TBI, particularly on *cis*- or *trans*-mechanisms. Moreover, linking the expression of mRNAs, we consider that these altered lncRNAs also affect their presentation, and it is worth investigating the relationships between them.

## Conclusion

Microarray was performed in the tissues of sham and TBI. We identified vital DE lncRNA and DE mRNAs between sham and TBI groups. Bioinformatics analysis identified the potential functions of DE mRNAs, and the regulatory network was constructed. Through prediction analysis, lncRNAs *trans*- and *cis*-regulatory functions were investigated. As a result, we suggested that multiple lncRNA–mRNA networks may be involved in key signaling pathways throughout inflammation and immune response during TBI. This research provides novel bioinformatic insights into the role of lncRNA in the molecular mechanisms underlying TBI.

## Data Availability Statement

The original contributions presented in the study are included in the article/supplementary material, further inquiries can be directed to the corresponding authors.

## Ethics Statement

The animal study was reviewed and approved by the Institutional Ethical Committee approved this study of the Central South University.

## Author Contributions

YWa and TT contributed to the design of the study. ZY wrote the manuscript and performed the statistical analysis. ZY, XL, WL, and YWu performed the experiments. All authors were responsible for the final version manuscript.

## Conflict of Interest

The authors declare that the research was conducted in the absence of any commercial or financial relationships that could be construed as a potential conflict of interest.

## Publisher’s Note

All claims expressed in this article are solely those of the authors and do not necessarily represent those of their affiliated organizations, or those of the publisher, the editors and the reviewers. Any product that may be evaluated in this article, or claim that may be made by its manufacturer, is not guaranteed or endorsed by the publisher.

## Footnotes

1
https://metascape.org/gp/index.html

2
http://kobas.cbi.pku.edu.cn/kobas3

3
https://cytoscape.org/

4
www.microRNA.gr/LncBase

5
http://mirdb.org/
